# Incidence of nosocomial pneumonia in two intensive care units of a French University Hospital from 2016 to 2022 in the era of COVID-19 pandemic^☆^

**DOI:** 10.1016/j.infpip.2025.100463

**Published:** 2025-05-08

**Authors:** Sélilah Amour, Elisabetta Kuczewski, Elodie Marion, Laurent Argaud, Julien Crozon-Clauzel, Anne Claire Lukaszewicz, Philippe Vanhems, Nagham Khanafer

**Affiliations:** a Unité d’Epidémiologie et Biomarqueur de l'infection, Hôpital Edouard Herriot, Groupement Hospitalier Centre, Hospices Civils de Lyon, 69003 Lyon, France; b Public Health, Epidemiology and Evolutionary Ecology of Infectious Diseases (PHE3ID), Centre International de Recherche en Infectiologie (CIRI), Inserm, U1111, Université Claude Bernard Lyon 1, CNRS, UMR5308, ENS de Lyon, 69007 Lyon, France; c Unité d’Hygiène, Epidémiologie et Prévention, Hôpital Edouard Herriot, Groupement Hospitalier Centre, Hospices Civils de Lyon, 69003 Lyon, France; d Medical Intensive Care Unit, Hôpital Edouard Herriot, Hospices Civils de Lyon, 69003 Lyon, France; e Anaesthesia and Critical Care Medicine Department, Hôpital Edouard Herriot, Hospices Civils de Lyon, 69002 Lyon, France

**Keywords:** Intensive care units, COVID-19 pandemic, Hospital-acquired pneumonia, Ventilator-acquired pneumonia, *Enterobacteriaceae*

## Abstract

**Background:**

Hospital-Acquired Pneumonia (HAP) are common in intensive care units (ICUs). The COVID-19 pandemic led to a global increase in healthcare-associated infections (HAI) among ICU patients. The aim of this study was to evaluate the trends in HAP incidence over a seven-year period of surveillance in two ICUs at a French University Hospital, and to assess the impact of COVID-19 (as well as the associated bacterial ecology).

**Methods:**

A prospective surveillance of HAI in ICUs was conducted during the 1^st^ quarter of each year between 2016 and 2022 (2020: reference year). Socio-demographic, clinical and bacteriological data were collected and the incidence of HAP was calculated. Poisson regressions were done and crude and adjusted incidence rate ratio were calculated.

**Results:**

1,797 patients were included, with 61.3% of male and a median age of 67 years. The median duration of intubation was 4 days (7 days in 2021 and 5 days in 2022). The proportion of COVID-19 patients was 45.7% in 2021 and 24.1% in 2022. Compared to 2020, the incidence of HAP increased in both 2021 [cIRR: 2.34 (95%CI: 1.30–4.23) and aIRR: 2.26 (95%CI: 1.25–4.08)] and 2022 [cIRR: 1.79 (95%CI: 0.97–3.32) and aIRR: 1.66 (95%CI: 0.90–3.07)]. The most commonly identified microorganisms were Enterobacteriaceae (42.4%), with a significantly higher incidence of HAP due to Enterobacteriaceae in COVID-19 patients.

**Conclusions:**

These results indicate an increase of HAP incidence in 2021 and 2022, mainly caused by *Enterobacteriaceae* in COVID-19 patients. This trend needs to be confirmed or refuted in the post-pandemic era.

## Introduction

Healthcare-associated infections (HAI), particularly hospital-acquired pneumonia (HAP), are a well-known problem for patients in intensive care units (ICUs), which often lead to significant morbidity, mortality, and costs [1]. The critical state of these patients, frequently exposed to invasive devices, such as intubation tubes, urinary catheters and central venous catheters, are the main risk factors of HAP. Furthermore, a prolonged intubation can be complicated by ventilator-acquired pneumonia (VAP), which in turn increases the duration of mechanical ventilation and ICU length of stay [2].

Respiratory viruses, such as Influenza A/H1N1, at admission, represent an additional risk factor of complications in ICU patients, increasing the risk of VAP [3]. This increased risk is mainly due to the dysregulation of lung immune defences [4], or gastric and oropharyngeal colonization [5].

In particular, during the COVID-19 pandemic (March 2020–May 2023, WHO), ICU patients were exposed to additional risk factors. Firstly, VAP has been described as a complication of COVID-19 pneumonitis and the incidence of VAP among COVID-19 patients appears to be higher (45.4% - > 50%) than in other viral pneumonia (23–40%) [[6], [7], [8]]. Recent data from the REA-REZO network suggest that the mortality due to VAP is higher in patients mechanically ventilated for severe COVID-19 compared to the general ICU population [9]. In addition, data from a multicentre cohort study showed that the 30-day fatality of VAP in critically ill patients with COVID-19 was as high as 46% [10].

The increased risk of nosocomial infection among COVID-19 hypoxemic patients is proportional to the severity of their condition [11] and the extent of care they require. These patients typically necessitate prolonged mechanical ventilation or extracorporeal membrane oxygenation (ECMO) [12,13], as well as more frequent prone positioning [14,15]. Immunomodulatory therapies, such as corticosteroids, are also commonly administered [16].

Moreover, the stressful conditions in ICUs during the peak of the COVID-19 pandemic may have contributed to the increased incidence of HAIs. These units experienced overcrowding, work overload, staff shortages, involvement of personnel not usually dedicated to ICUs, and a shortage of personal protective equipment [17]. All of these factors may have affected the application of infection control measures and VAP prevention recommendations [18], potentially leading to cross-contamination. On the other hand, the heightened awareness of the risk of infection among healthcare workers during the pandemic may have increased vigilance in preventing the risk of infection.

The objective of this study was to analyse trends in the rate of HAP in two ICUs of a French university hospital from 2016 to 2022, with a focus on the impact of COVID-19 on these nosocomial infections and on their bacterial ecology.

## Methods

### Study design and population

The study was carried out in two ICU wards (respectively a polyvalent ICU with surgical and medical activities and a medical unit) of Edouard Herriot Hospital, a 1160-bed university hospital in Lyon, France. The polyvalent ICU provided specialized care for patients requiring urgent renal support, and it served as an expert center for extracorporeal purification in septic shock patients, as well as providing post-transplant care for kidney and kidney-pancreas recipients. The medical ICU treated a wide range of life-threatening conditions, such as respiratory failure, circulatory failure (shock), cardiac arrest, and coma, irrespective of the cause. The study included adult patients who had been hospitalized in these ICU's for at least 48 hours during the 1^st^ quarter of the years 2016–2022. The inclusion date was the date of discharge. Patients were followed until they were discharged from the ICU or deceased. For each patient, a standardized data sheet was completed prospectively, including sociodemographic, clinical and microbiological data.

### Ethics and consent

Data were obtained from the REA-REZO national surveillance network for HAI in ICUs in France [19]. The REA-REZO database was approved by the National Data Protection Commission (*Commission nationale de l'informatique et des libertés*, Number 919149) and the institutional review board (CPP SUD EST - IRB 00009118). All patients included in this database received information about the use of their personal data for research purposes and were given the opportunity to refuse it (during or after their intensive care stay, and in case the patient was unable refuse participation, family or close relatives could refuse the use of data on behalf of the patient). According to French law, written informed consent was not required.

### Definitions of HAP and VAP

Pneumonia was defined using a combination of clinical, radiological, and laboratory criteria (Supplementary Table S1) [20]. A HAP was defined as a pneumonia occurring 48 hours or more after hospital admission, while a VAP was considered to be a pneumonia that occurred in a patient who had been mechanically ventilated for more than 48 hours and no more than two days after extubation. Only the first HAP or VAP was considered for analysis.

### Statistical analysis

A descriptive analysis reports the characteristics of included patients. Categorical variables are described with absolute and relative frequencies, which are compared using the Chi-square test or Fisher exact test, as appropriate. Continuous variables are described as the mean (± Standard Deviation (SD)) and are compared using the Mann-Whitney U-test, Wilcoxon test or Kruskal-Wallis test. The incidence of HAP was calculated per 100 hospitalised patients (attack rate or cumulative incidence rate) and for 1,000 days of hospitalisation (incidence rate or density). Similarly, the incidence of VAP was calculated per 100 intubated patients (attack rate or cumulative incidence rate) and for 1,000 days of intubation (incidence rate or density). The results are presented with the 95% confidence interval (95% CI). Poisson regressions were used to assess the temporal trends of the crude and adjusted incidence rate ratios (IRRs), with age, gender, ICU, and SAPS II severity score used as adjustment factors. The year 2020 was chosen as the reference year, as COVID-19 PCR tests were not yet completely available in the first quarter of that year. Additionally, the incidence was calculated separately for the three most common microorganisms implicated in HAP. *P*-values < 5% are considered significant, and all tests are 2-tailled. Data analysis is performed using Stata SE version 17 (StataCorp. LP).

## Results

### Population characteristics

A total of 1,797 patients were included in the study, with a mean of age of 64.5 years (±16.0) and a male-to-female ratio of 1.6 (Table I). The mean length of stay (LOS) increased significantly from 7.4 days in 2016 to 9.8 days in 2022 (*P*=0.04). However, the mean SAPS II score and the proportion of intubated patients remained constant throughout the study period. Furthermore, the average duration of intubation increased significantly from 7.6 days in 2016 to 10.5 days in 2022 (*P*<0.001).

**Table I:** Characteristic of patients admitted in two ICU departments at Edouard Herriot Hospital, 1^st^ quarter 2016–2022 (n=1,797)

	2016 N=273	2017 N=272	2018 N=274	2019 N=283	2020 N=286	2021 N=210	2022 N=199
Sex – n (%)							
Male	182 (66.7)	149 (54.8)	164 (59.9)	179 (63.2)	180 (62.9)	120 (57.1)	128 (64.3)
Female	91 (33.3)	123 (45.2)	110 (40.1)	104 (36.8)	106 (37.1)	90 (42.9)	71 (35.7)
Sex-ratio (M/F)	2.0	1.2	1.5	1.7	1.7	1.3	1.8
Age (year) – Mean (±SD)	64.1 (±17.0)	67.0 (±15.9)	65.7 (±15.0)	65.5 (±16.0)	64.1 (±16.6)	63.9 (±13.4)	59.9 (±16.9)
SAPS II - Mean (±SD)	50.3 (±20.1)	52.4 (±18.1)	52.6 (±18.7)	51.3 (±18.5)	48.9 (±18.6)	46.5 (±18.5)	49.9 (±19.6)
In-hospital mortality- n (%)	41 (15.0)	50 (18.4)	46 (16.8)	42 (14.8)	49 (17.1)	29 (13.8)	40 (20.1)
Antibiotics at admission – n (%)	212 (78.5)	214/263 (81.4)	222/269 (82.5)	222/282 (78.7)	229 (80.1)	174 (82.9)	147 (73.9)
Immunosuppression – n (%)	77 (28.2)	47 (17.3)	34 (12.6)	38 (13.4)	16 (5.6)	25 (11.9)	34 (17.8)
Including <500 neutrophils/mm^3^	0 (0.0)	0 (0.0)	1 (0.4)	1 (0.4)	4 (1.4)	3 (3.1)	6 (3.1)
Cumulative patient days (PD) of hospitalisation	2013	1716	1874	1789	1884	1944	1949
Duration of hospitalisation (day) – Mean (±SD)	7.4 (±11.0)	6.3 (±7.5)	6.8 (±8.4)	6.3 (±7.3)	6.6 (±6.5)	9.3 (±12.2)	9.8 (±13.4)
Invasive device exposure – n (%)							
Intubation	149 (54.6)	153 (56.3)	155 (56.6)	154 (54.4)	149 (52.1)	123 (58.6)	120 (60.3)
Central venous catheter	201 (73.6)	209 (77.1)	209 (76.3)	191 (67.5)	189 (66.1)	137 (65.2)	131 (65.8)
Exposure duration (day) – Mean (±SD)							
Intubation	7.6 (±13.0)	5.8 (±7.2)	6.7 (±9.0)	5.6 (±6.5)	6.7 (±6.7)	10.6 (±13.2)	10.5 (±13.8)
Central venous catheter	7.4 (±11.0)	6.3 (±7.5)	6.8 (±8.4)	6.3 (±7.3)	6.6 (±6.5)	9,3 (±12.2)	9.8 (±13.4)

Only the first nosocomial infection of each patient was included in the rate calculations.

SAPS II, Simplified Acute Physiology Score II.

SD, Standard Deviation.

### Description of SARS-CoV-2 cases

The prevalence of SARS-CoV-2 confirmed by RT-PCR was 47% (n=96) in 2021, and 24% (n=48) in 2022. The mean LOS was significantly longer for COVID-19 than for non COVID-19 patients during both years. In 2021, the LOS of COVID-19 patients was 12.4 days versus (*vs*) 6.7 days for non-COVID-19 patients (*P*<0.001), and in 2022, it was 16.0 days in COVID-19 patients *vs* 7.8 days for non-COVID-19 patients (*P*<0.001). The intubation rate was significantly lower among COVID-19 patients than in non-COVID-19 patients in 2021 (48% *vs* 68% respectively, *P=*0.004), but no significant statistical difference was detected in 2022 (60% in both groups). The mean duration of intubation was significantly longer among COVID-19 patients, reaching 18.4 days in 2021 and 17.8 days in 2022, compared to non COVID-19 patients who experienced durations of 6.1 days in 2021 and 8.2 days in 2022 (*P*<0.001 for both years). The reintubation rate was higher among COVID-19 patients than among non COVID-19 patients in both years (15% and 17% respectively *vs* 8% in both years, non-significant difference, *P=*0.223 and *P=*0.147 respectively in 2021 and 2022).

### Hospital-acquired pneumonia and ventilator acquired pneumonia

**Figure 1:**
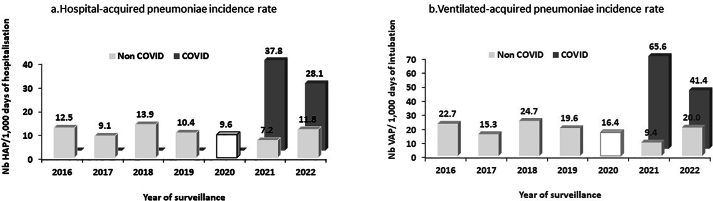
Hospital-Acquired Pneumonia incidence rate and Ventilated-Acquired Pneumonia incidence rate according to year of surveillance, 1^st^ quarter 2016–2022 (a: n=1797; b: n=1003).

The attack rate of HAP per 100 patients showed a significant increase from 7.3 in 2016 to 12.6 in 2022 (*P*<0.001) (Supplementary Table S2). This increase was particularly pronounced among COVID-19 patients when compared to non-COVID-19 patients, with rates of 26.0 (*vs* 4.6) per 100 patients in 2021 (*P*<0.001) and 29.2 (*vs* 7.4) per 100 patients in 2022 (*P*<0.001)). Additionally, the incidence rate of HAP per 1,000 days of hospitalisation significantly increased from 12.5 in 2016 to 17.3 in 2022 (*P*<0.001) (Supplementary Table S2). Similarly, the incidence rate of HAP was significantly higher among COVID-19 patients than in non-COVID-19 patients, with rates of 37.8 (*vs* 7.2) per 1,000 days of hospitalization in 2021 (*P*<0.001) and 28.1 (*vs* 11.8) per 1,000 days of hospitalization in 2022 (*P*=0.03 (Figure 1a).

The attack rate of VAP per 100 intubated patients increased significantly from 12.1 in 2016 to 20.0 in 2022 (*P*<0.001) (Supplementary Table S2). Notably, the rate among COVID-19 patients was significantly higher than that of non COVID-19 patients (54.3 *vs* 5.4 in 2021 (*P*<0.001) and 44.8 *vs* 12.4 in 2022 (*P*<0.001)]. Moreover, the incidence rate of VAP per 1,000 days of intubation increased significantly from 22.7 in 2016 to 27.6 in 2022 (*P*=0.08) (Supplementary Table S2). This rate was significantly higher in COVID-19 patients than that of non COVID-19 patients (65.6 *vs* 9.4 in 2021 (*P*<10^−4^) and 41.4 *vs* 20.0 in 2022 (*P*=0.119)) (Figure 1b).

### Crude and adjusted IRR HAP and VAP

**Figure 2:**
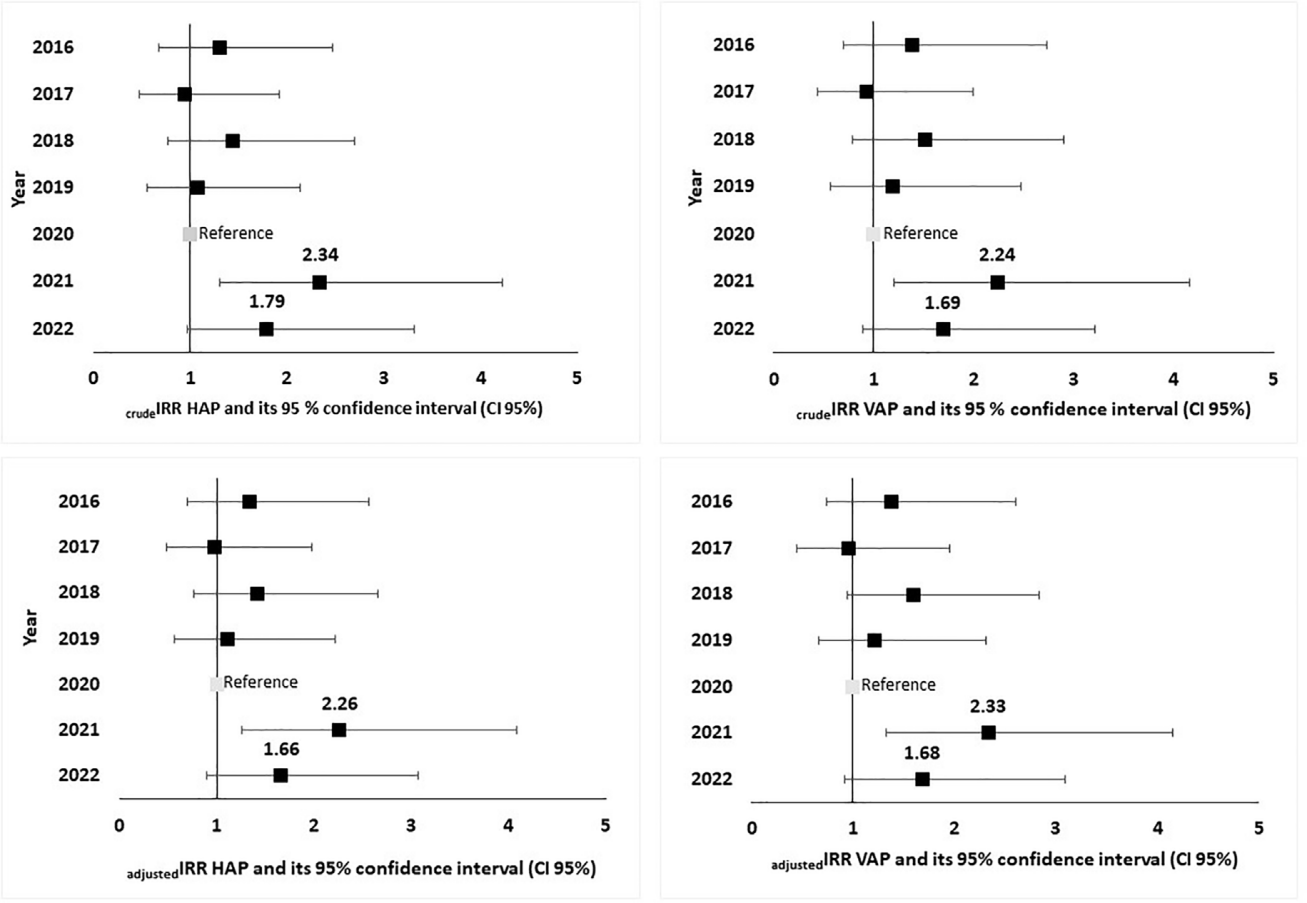
Crude and adjusted incidence rate ratio of hospital-acquired pneumonia and ventilated-acquired pneumoniae according to year of surveillance, 1^st^ quarter 2016–2022 (IRR HAP: n=1797; IRR VAP: n=1003).

The crude IRR for HAP was 2.34 (CI95%: 1.30–4.22) in 2021 and 1.79 (CI95%: 0.97–3.22) in 2022. Similarly, the crude IRR for VAP was 2.24 (CI95%: 1.20–4.16) in 2021 and 1.69 (CI95%: 0.89–3.22) in 2022. After adjusting for confounding factors, the IRRs for HAP were 2.26 (CI95%: 1.25–4.08) in 2021 and 1.66 (CI95%: 0.90–3.07) in 2022. The adjusted IRRs for VAP was 2.33 (CI95%: 1.25–4.32) in 2021 and 1.68 (CI95%: 0.88–3.19) in 2022 (Figure 2).

### HAP incidence rate by microorganisms

**Figure 3:**
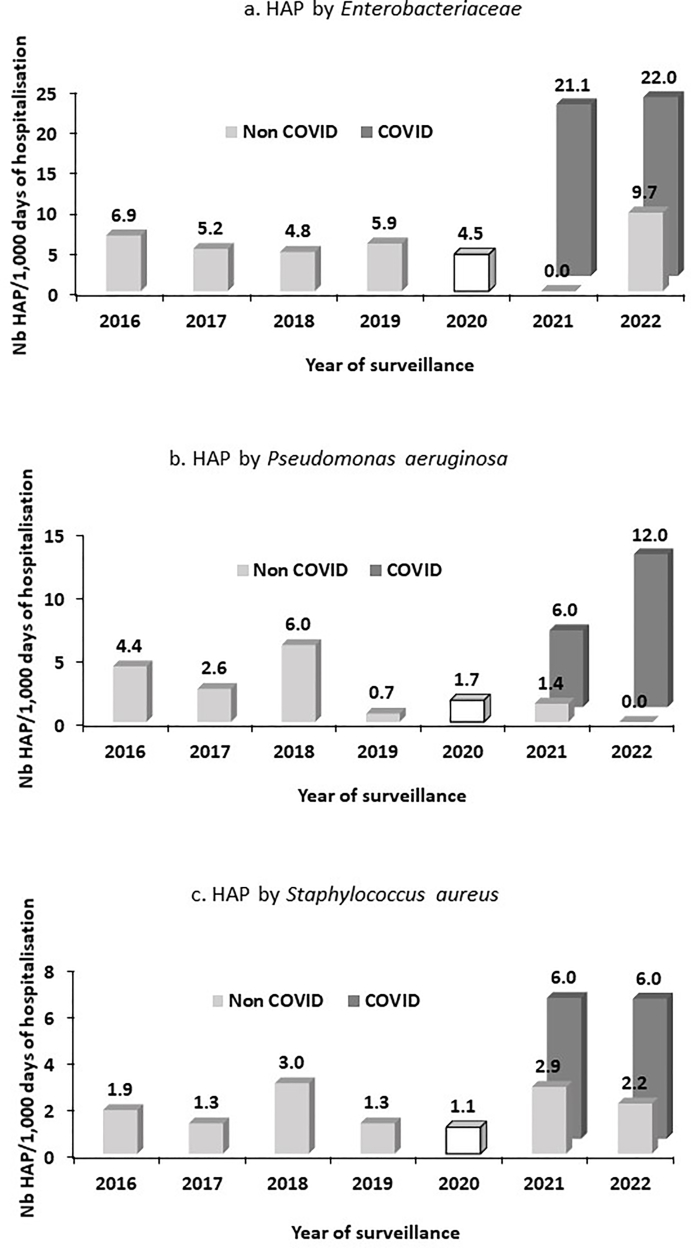
Hospital-acquired pneumoniae incidence rate according to year of surveillance and by microorganisms, 1^st^ quarter 2016–2022 (n=1797).

In all, we found 184 microorganisms responsible for pneumonia. They are detailed in the supplementary Table S3. The incidence rate of HAP was calculated based on the most commonly identified microorganisms associated with these infections: Enterobacteriaceae, *Pseudomonas aeruginosa* and *Staphylococcus aureus* (Figure 3). Specifically, the incidence of Enterobacteriaceae among COVID-19 patients in 2021 was 21.1 (CI95%: 11.6–35.5) and 22.0 (CI95%: 11.0–39.4) in 2022. In contrast, the incidence rate among non COVID-19 patients, was zero (CI95%: undefined-5.3) in 2021 and 9.7 (CI95%: 4.4–18.4) in 2022 (*P*<0.001 and *P*=0.11 respectively). The incidence rate of *P*. *aeruginosa* among COVID-19 patients was 6.0 (CI95%: 1.6–15.5) in 2021 and 12.0 (CI95%: 4.4–26.2) in 2022, while it was 1.4 (CI95%: 0.0–8.0) and 0 (undefined-3.9) in non COVID-19 patients (*P=*0.34 and *P=*0.003, respectively). Lastly, the incidence of *S*. *aureus* among COVID-19 patients was 6.0 (CI95%: 1.6–15.5) in 2021 and 6.0 (CI95%: 1.2–17.6) in 2022, and it was 2.9 (CI95%: 0.3–10.3) and 2.2 (CI95%: 0.2–7.8) among non COVID-19 patients (*P*=0.64 and *P*=0.47, respectively).

In terms of antimicrobial resistance, approximately 38.7% of Enterobacteriaceae species exhibited resistance to third generation cephalosporins (C3G), while 1.3% were resistant to a carbapenem. Additionally, 26.9% of *S*. *aureus* isolates were found to be methicillin-resistant, and 28.6% and 34.3% of *P*. *aeruginosa* isolates were resistant to a carbapenem and ceftazidime, respectively (Supplementary Figures S1 and S2).

### Patient prognosis

The in-hospital mortality rate was similar for COVID-19 (13.5%) and non COVID-19 patients (13.8%) in 2021. However, in 202, this rate was higher for COVID-19 patients (27.1%) than for non COVID-19 patients (18.2%). The intubation rate for COVID-19 patients who died was 100% in 2021, while the rate for non COVID-19 patients who died was 86.7%. In 2022, the intubation rates were similar for both populations (84.6% *vs* 85.2% respectively). In 2021, 69.2% of COVID-19 patients who died had pneumonia (55% by Enterobacteriaceae), *vs* 6.7% of non COVID-19 (none by Enterobacteriaceae). In 2022, 30.8% of COVID-19 patients who died had pneumonia (100% caused by Enterobacteriaceae) compared to 11.1% of non COVID-19 patients, with 66.7% caused by Enterobacteriaceae.

## Discussion

The results of this study show a rise in the incidence of HAP and VAP coinciding with the COVID-19 pandemic in France. This increase was limited to COVID-19 patients, suggesting that it was not due to extrinsic reasons such as ICU stress conditions and work overload caused by the pandemic [21]. Instead, the rise in incidence is likely due to the particular profile of COVID-19 patients, such as hypoxemic conditions, lung damage [11], long-term mechanical ventilation or ECMO [12,13], more frequent use of prone positioning [14,15], and the use of immunomodulatory therapies, like corticosteroids [16]. The multivariate analysis showed that age, gender, ICU type and SAPS II severity score did not contribute to the observed increase in HAP incidence.

In addition to the increase of incidence of HAP and VAP among COVID-19 patients, we found that the LOS of COVID-19 patients was significantly longer than for non COVID-19 patients, primarily due to the former's greater clinical severity. Additionally, COVID-19 patients experienced extended durations of intubation, which were roughly three times longer in 2021 and two times longer in 2022 than those of non COVID-19 patients. Although the re-intubation rate of COVID-19 patients was not significantly higher, the higher number of extubation failures for COVID-19 patients suggests a potential need for more aggressive interventions. Notably, the intubation rate for COVID-19 patients in 2021 was significantly lower (by approximately 30%) than that of non COVID-19 patients, indicating a potential alternative approach for COVID-19 patients in that year, such as the use of a high flow nasal oxygen cannula and non-invasive ventilation [22,23].

Our results indicated a rise in the incidence of HAP infections caused by Enterobacteriaceae, particularly those resistant to C3G, in COVID-19 patients since the beginning of the pandemic. It has been shown that COVID-19 patients have a higher risk of developing severe, nosocomial infections caused by carbapenemase producing Enterobacteriacae, which often occur in critically-ill patients and are associated with a high mortality rate [24]. However, this was not observed in our hospital. Upon examining the mortality rate, it was observed that a significant proportion, more than half, of the cases of nosocomial pneumonia in 2021 and all cases in 2022 were caused by Enterobacteriaceae.

The COVID-19 pandemic has had a considerable impact on the epidemiology of respiratory viruses, which has been influenced by non-pharmaceutical interventions and potential viral interactions [[25], [26], [27]]. During this pandemic, the peak of community seasonal influenza was significantly reduced [28,29]. This was also evident In Lyon university hospital during the first quarter of 2021, where no influenza cases were reported in the internal weekly epidemiological bulletin. This aligns with the absence of critical influenza cases in the ICU during the same period. In 2021, the incidence densities of HAP and VAP among non COVID-19 patients were respectively 0.7 times and 0.5 times smaller than the average values for the period 2016–2019. Furthermore, the re-intubation rate in 2021 was lower than in the previous period. In summary, the first quarter of 2021 was similar to the third one, as both periods were free of influenza's influence (data not shown).

Some limitations must be addressed. First, the findings of this study are dependent on data collected from just two ICUs. Second, the data was only gathered during the first quarter of each year. Lastly, data for 2023 are not yet available to determine whether the increasing trends in the incidence of HAP and VAP persist. However, this study allowed us to describe the trends of nosocomial pneumonia rates, including their microbial ecology, during the first quarter from 2016 to 2022, when respiratory viruses are prevalent. Additionally, the collected microbiological data highlighted a local rise in cases of Enterobacteriaceae pneumonia during the pandemic.

## Conclusions

It will be noteworthy to examine the incidence of HAP and VAP during the first quarters of 2023 and 2024 to assess the impact of the resurgence of influenza on these complications and to evaluate the potential shift to a seasonal pattern of SARS-CoV-2 transmission. Furthermore, it will be necessary to estimate the prevalence of HAP attributed to Enterobacteriaceae in the post-pandemic era.

## Credit author statement

Amour, Kuczewski, Khanafer and Vanhems had full access to all of the data in the study and take responsibility for the integrity of the data and the accuracy of the data analysis.

*Concept and design:* Amour, Kuczewski, Khanafer and Vanhems.

*Acquisition, analysis, or interpretation of data:* All Authors.

*Drafting of the manuscript:* Amour, Kuczewski and Khanafer.

*Critical revision of the manuscript for important intellectual content:* All Authors.

*Statistical analysis:* Amour, Kuczewski and Khanafer.

*Obtained funding:* Not applicable.

*Administrative, technical, or material support:* Amour, Kuczewski, Khanafer and Vanhems.

*Supervision:* Vanhems.

Read and approved the final manuscript: All authors.

## Funding/support

None.

## Conflict of interest disclosures

All authors have completed and submitted the ICMJE Form for Disclosure of Potential Conflicts of Interest. All authors reported no conflict of interest related to this study.
