# Supplementary material for: Incidence of nosocomial pneumonia in two intensive care units of a French University Hospital from 2016 to 2022 in the era of COVID-19 pandemic
Source: Infect Prev Pract. 2025 May 8;7(2):100463. doi: 10.1016/j.infpip.2025.100463 (PMC12147891; doi:10.1016/j.infpip.2025.100463)
Supplement: Multimedia component 1 [file mmc1.docx]

**Supplementary material**

**Table template**

**Table S1 -** Definition of pneumonia according to ECDC definition

**Table S2 -** Hospital-acquired pneumonia (HAP) and Ventilated-acquired pneumonia (VAP) according to year of surveillance, 2016-2022 (n=1,797)

**Table S3 -** Microorganisms detected among pneumonia cases (n=184)

**Figure template**

**Figure S1 -** HAP incidence rate by microorganisms and by antibiotic resistance

**Figure S2 -** Resistance profile of the three most frequent bacterial species

**Table S1 –** Definition of pneumonia according to ECDC definition^1^

| **X-ray** | Two or more serial chest X-rays or CT-scans with a suggestive image of pneumonia for patients with underlying cardiac or pulmonary disease* (in patients without underlying cardiac or pulmonary disease, one definitive chest Xray or CT-scan is sufficient). |
| --- | --- |
| **And at least one of the following:** | |
| **Symptoms** | Fever > 38 °C with no other cause |
|  | Leukopenia (< 4 000 WBC/mm3) or leucocytosis (≥ 12 000 WBC/mm3). |
|  | **And at least one of the following (or at least two, if clinical pneumonia only = PN 4 and PN 5):** |
|  | - new onset of purulent sputum, or change in character of sputum (colour, odour, quantity, consistency) |
|  | - cough or dyspnea or tachypnea |
|  | - suggestive auscultation (rales or bronchial breath sounds), rhonchi, wheezing |
|  | - worsening gas exchange (e.g. O2 desaturation or increased oxygen requirements or increased ventilation demand) |
| **And** | |
| **according to the used diagnostic method:** | |
| **Microbiology** | **A - Bacteriologic diagnostic performed by:** |
|  | Positive quantitative culture from minimally contaminated LRT specimen **(PN 1)** |
|  | - broncho-alveolar lavage (BAL) with a threshold of ≥ 104 colony forming units (CFU)/ml or ≥ 5% of BAL obtained cells contain intracellular bacteria on direct microscopic exam (classified on the diagnostic category BAL) |
|  | - protected brush (PB Wimberley) with a threshold of ≥ 103 CFU/ml |
|  | - distal protected aspirate (DPA) with a threshold of ≥ 103 CFU/ml. |
|  | Positive quantitative culture from possibly contaminated LRT specimen **(PN 2)** |
|  | - Quantitative culture of LRT specimen (e.g. endotracheal aspirate) with a threshold of 106 CFU/ml. |
|  | **B - Alternative microbiology methods (PN 3)** |
|  | - positive blood culture not related to another source of infection |
|  | - positive growth in culture of pleural fluid |
|  | - pleural or pulmonary abscess with positive needle aspiration |
|  | - histologic pulmonary exam shows evidence of pneumonia |
|  | - positive exams for pneumonia with virus or particular germs (e.g. *Legionella, Aspergillus, mycobacteria, mycoplasma,* *Pneumocystis jiroveci* [previously *P.carinii*]): |
|  | - positive detection of viral antigen or antibody from respiratory secretions (e.g. EIA, FAMA, shell vial assay, PCR) |
|  | - positive direct exam or positive culture from bronchial secretions or tissue |
|  | - seroconversion (example: *influenza viruses, Legionella, Chlamydia*) |
|  | - detection of antigens in urine (*Legionella*). |
|  | **C - Others** |
|  | Positive sputum culture or non-quantitative LRT specimen culture **(PN 4)** |
|  | No positive microbiology **(PN 5)** |

**^1^**[**https://www.ecdc.europa.eu/sites/default/files/documents/HAI-Net-ICU-protocol-v2.2_0.pdf**](https://www.ecdc.europa.eu/sites/default/files/documents/HAI-Net-ICU-protocol-v2.2_0.pdf)

**Notes:**

- PN 1 and PN 2 criteria were validated without previous antimicrobial therapy. However, this does not exclude the diagnosis of PN 1 or PN 2 in the case of previous antimicrobial use
- *In case recent chest X-rays are available for patients with underlying cardiac or pulmonary disease, one definitive chest X-ray or CT-scan during the current ICU stay may be sufficient.

**Table S2 -** Hospital-acquired pneumonia (HAP) and Ventilated-acquired pneumonia (VAP) according to year of surveillance, 2016-2022 (n=1,797)

|  | **2016**  **N=273** | **2017**  **N=272** | **2018**  **N=274** | **2019**  **N=283** | **2020**  **N=286** | **2021**  **N=210** | **2022**  **N=199** |
| --- | --- | --- | --- | --- | --- | --- | --- |
| **Attack rate (95%CI)** |  |  |  |  |  |  |  |
| HAP (/100 patients) | 7.3 (4.6-11.1) | 5.1 (2.9-8.4) | 8.4 (5.5-12.4) | 5.7 (3.3-9.0) | 5.9 (3.6-9.3) | 14.8 (10.2-20.7) | 12.6 (8.3-18.3) |
| VAP (/100 intubated patients) | 12.1 (7.4-18.7) | 7.8 (4.3-13.3) | 14.2 (9.1-21.1) | 9.1 (5.2-14.9) | 10.1 (5.9-16.2) | 24.4 (16.8-34.4) | 20.0 (13.1-29.3) |
| **Number (N)** |  |  |  |  |  |  |  |
| Number of HAP | 20 | 14 | 23 | 16 | 17 | 31 | 25 |
| Number of VAP | 18 | 12 | 22 | 14 | 15 | 30 | 24 |
| **Duration (days)** |  |  |  |  |  |  |  |
| Interval from ICU admission to HAP |  |  |  |  |  |  |  |
| Mean (±SD) | 5.9 (±5.1) | 5.7 (±5.7) | 6.0 (±6.6) | 5.4 (±4.7) | 6.2 (±5.9) | 6.5 (±6.3) | 7.3 (±7.3) |
| Total | 1603 | 1539 | 1655 | 1534 | 1765 | 1375 | 1447 |
| Interval from MV onset to VAP |  |  |  |  |  |  |  |
| Mean (±SD) | 5.4 (±5.4) | 5.2 (±5.9) | 5.8 (±7.7) | 4.7 (±4.8) | 6.2 (±8.9) | 6.7 (±6.7) | 7.3 (±7.6) |
| Total | 794 | 784 | 889 | 713 | 917 | 818 | 870 |
| **Incidence (95%CI)** |  |  |  |  |  |  |  |
| HAP (/1,000 days of hospitalisation) | 12.5 (7.8-18.9) | 9.1 (5.2-14.9) | 13.9 (9.0-20.5) | 10.4 (6.2-16.6) | 9.6 (5.8-15.1) | 22.5 (15.6-31.6) | 17.3 (11.4-25.1) |
| VAP (/1,000 days of intubation) | 22.7 (13.9-35.1) | 15.3 (8.3-26.0) | 24.7 (15.9-36.9) | 19.6 (11.2-32.2) | 16.4 (9.5-26.4) | 36.7 (25.2-51.7) | 27.6 (18.1-40.4) |

95%CI: 95% Confidence Interval; ICU: Intensive Care Unit; SD: Standard Deviation; MV: Mechanical Ventilation

**Table S3 –** Microorganisms detected among Hospital-acquired pneumonia cases (HAP) (n=184)

| **Category** | **Name** | **2016** | **2017** | **2018** | **2019** | **2020** | **2021** | **2022** | **Total** |
| --- | --- | --- | --- | --- | --- | --- | --- | --- | --- |
| Gram - bacilli non-enterobacteria | *Achromobacter* | 0 | 0 | 0 | 1 | 0 | 0 | 0 | 1 |
| Gram - bacilli non-enterobacteria | *Acinetobacter other species* | 0 | 0 | 0 | 0 | 1 | 0 | 0 | 1 |
| Gram - bacilli non-enterobacteria | *Haemophilus* | 0 | 1 | 0 | 0 | 1 | 3 | 1 | 6 |
| Gram - bacilli non-enterobacteria | *Pseudomonas aeruginosa* | 7 | 4 | 10 | 1 | 3 | 5 | 6 | 36 |
| Gram - bacilli non-enterobacteria | *Stenotrophomonas maltophilia* | 0 | 0 | 0 | 3 | 1 | 2 | 0 | 6 |
| **Total (n)** |  | **7** | **5** | **10** | **5** | **6** | **10** | **7** | **50** |
| **Total (n/N)** |  | **32%** | **31%** | **38%** | **25%** | **27%** | **25%** | **18%** | **27%** |
| Fungi / parasites | *Aspergillus other species* | 0 | 0 | 0 | 0 | 0 | 0 | 1 | 1 |
| Fungi / parasites | *Candida albicans* | 0 | 0 | 0 | 0 | 1 | 0 | 1 | 2 |
| Fungi / parasites | *Candida krusei* | 0 | 0 | 0 | 1 | 0 | 0 | 0 | 1 |
| **Total** |  | **0** | **0** | **0** | **1** | **1** | **0** | **2** | **4** |
| **Total (n/N)** |  | **0%** | **0%** | **0%** | **5%** | **5%** | **0%** | **5%** | **2%** |
| Cocci Gram - | *Moraxella* | 0 | 0 | 1 | 0 | 0 | 0 | 1 | 2 |
| **Total** |  | **0** | **0** | **1** | **0** | **0** | **0** | **1** | **2** |
| **Total (n/N)** |  | **0%** | **0%** | **4%** | **0%** | **0%** | **0%** | **3%** | **1%** |
| Cocci Gram + | *Staphylococcus aureus* | 3 | 2 | 5 | 2 | 2 | 8 | 4 | 26 |
| Cocci Gram + | *Staphylococcus epidermidis* | 1 | 0 | 0 | 0 | 0 | 1 | 0 | 2 |
| Cocci Gram + | *Staphylococcus haemolyticus* | 0 | 0 | 0 | 1 | 0 | 0 | 0 | 1 |
| Cocci Gram + | *Streptococcus other species* | 0 | 0 | 0 | 1 | 0 | 0 | 0 | 1 |
| Cocci Gram + | *Streptococcus (viridans) non-groupable* | 0 | 0 | 0 | 0 | 0 | 0 | 1 | 1 |
| Cocci Gram + | *Streptococcus pneumoniae* | 0 | 1 | 0 | 1 | 2 | 1 | 2 | 7 |
| **Total** |  | **4** | **3** | **5** | **5** | **4** | **10** | **7** | **38** |
| **Total (n/N)** |  | **18%** | **19%** | **19%** | **25%** | **18%** | **25%** | **18%** | **21%** |
| *Enterobacteriaceae* | *Citrobacter freundii* | 0 | 1 | 0 | 0 | 0 | 1 | 2 | 4 |
| *Enterobacteriaceae* | *Citrobacter koseri* | 0 | 1 | 0 | 0 | 0 | 1 | 2 | 4 |
| *Enterobacteriaceae* | *Enterobacter aerogenes* | 1 | 1 | 0 | 0 | 3 | 2 | 2 | 9 |
| *Enterobacteriaceae* | *Enterobacter cloacae* | 1 | 2 | 3 | 1 | 0 | 1 | 3 | 11 |
| *Enterobacteriaceae* | *Escherichia coli* | 4 | 1 | 2 | 3 | 3 | 0 | 3 | 16 |
| *Enterobacteriaceae* | *Hafnia* | 1 | 1 | 0 | 0 | 1 | 0 | 1 | 4 |
| *Enterobacteriaceae* | *Klebsiella oxytoxa* | 0 | 0 | 0 | 1 | 0 | 0 | 0 | 1 |
| *Enterobacteriaceae* | *Klebsiella pneumoniae* | 2 | 1 | 1 | 2 | 1 | 5 | 4 | 16 |
| *Enterobacteriaceae* | *Morganella* | 2 | 0 | 0 | 0 | 0 | 2 | 0 | 4 |
| *Enterobacteriaceae* | *Proteus other species* | 0 | 0 | 0 | 0 | 0 | 0 | 1 | 1 |
| *Enterobacteriaceae* | *Proteus mirabilis* | 0 | 0 | 0 | 1 | 0 | 0 | 2 | 3 |
| *Enterobacteriaceae* | *Serratia* | 0 | 0 | 2 | 1 | 0 | 2 | 0 | 5 |
| **Total** |  | **11** | **8** | **8** | **9** | **8** | **14** | **20** | **78** |
| **Total (n/N)** |  | **50%** | **50%** | **31%** | **45%** | **36%** | **35%** | **53%** | **42%** |
| Virus | *Herpes simplex Virus* | 0 | 0 | 0 | 0 | 0 | 0 | 1 | 1 |
| **Total** |  | **0** | **0** | **0** | **0** | **0** | **0** | **1** | **1** |
| **Total (n/N)** |  | **0%** | **0%** | **0%** | **0%** | **0%** | **0%** | **3%** | **1%** |
| Examination not performed |  | 0 | 0 | 2 | 0 | 3 | 5 | 0 | 10 |
| Not found or not searched |  | 0 | 0 | 0 | 0 | 0 | 1 | 0 | 1 |
| **Total** |  | 0 | 0 | 2 | 0 | 3 | 6 | 0 | 11 |
|  |  | 0% | 0% | 8% | 0% | 14% | 15% | 0% | 6% |
| **Total (N)** |  | **22** | **16** | **26** | **20** | **22** | **40** | **38** | **184** |
|  |  | **100%** | **100%** | **100%** | **100%** | **100%** | **100%** | **100%** | **100%** |

**Figure S1 –** Hospital-acquired pneumonia (HAP) incidence rate by microorganisms and by antibiotic resistance

**Figure S2 –** Resistance profile of the three most frequent bacterial species

**Fi**
